# Cysteamine inhibits lysosomal oxidation of low density lipoprotein in human macrophages and reduces atherosclerosis in mice

**DOI:** 10.1016/j.atherosclerosis.2019.09.019

**Published:** 2019-12

**Authors:** Yichuan Wen, Feroz Ahmad, Zahra Mohri, Peter D. Weinberg, David S. Leake

**Affiliations:** a School of Biological Sciences and Institute for Cardiovascular and Metabolic Research, University of Reading, Reading, Berkshire, United Kingdom; b Department of Bioengineering, Imperial College London, London, United Kingdom

**Keywords:** Atherosclerosis, Cysteamine, Foam cells, Lysosomes, Oxidation of LDL

## Abstract

**Background and aims:**

We have shown previously that low density lipoprotein (LDL) aggregated by vortexing is internalised by macrophages and oxidised by iron in lysosomes to form the advanced lipid/protein oxidation product ceroid. We have now used sphingomyelinase-aggregated LDL, a more pathophysiological form of aggregated LDL, to study lysosomal oxidation of LDL and its inhibition by antioxidants, including cysteamine (2-aminoethanethiol), which concentrates in lysosomes by several orders of magnitude. We have also investigated the effect of cysteamine on atherosclerosis in mice.

**Methods:**

LDL was incubated with sphingomyelinase, which increased its average particle diameter from 26 to 170 nm, and was then incubated for up to 7 days with human monocyte-derived macrophages. LDL receptor-deficient mice were fed a Western diet (19–22 per group) and some given cysteamine in their drinking water at a dose equivalent to that used in cystinosis patients. The extent of atherosclerosis in the aortic root and the rest of the aorta was measured.

**Results:**

Confocal microscopy revealed lipid accumulation in lysosomes in the cultured macrophages. Large amounts of ceroid were produced, which colocalised with the lysosomal marker LAMP2. The antioxidants cysteamine, butylated hydroxytoluene, amifostine and its active metabolite WR-1065, inhibited the production of ceroid. Cysteamine at concentrations well below those expected to be present in lysosomes inhibited the oxidation of LDL by iron ions at lysosomal pH (pH 4.5) for prolonged periods. Finally, we showed that the extent of atherosclerotic lesions in the aortic root and arch of mice was significantly reduced by cysteamine.

**Conclusions:**

These results support our hypothesis that lysosomal oxidation of LDL is important in atherosclerosis and hence antioxidant drugs that concentrate in lysosomes might provide a novel therapy for this disease.

## Highlights

• The drug cysteamine, which accumulates in lysosomes, inhibited the oxidation of LDL by iron at pH 4.5 (the pH of lysosomes).
• Cysteamine inhibited the lysosomal oxidation of LDL inside cultured macrophages.
• Cysteamine reduced atherosclerosis in LDL receptor knockout mice.
• These results support our hypothesis that lysosomal oxidation of LDL is important in atherosclerosis.
• Antioxidant drugs that concentrate in lysosomes might provide a novel therapy for this disease.

## Graphical abstract

**Figure:**
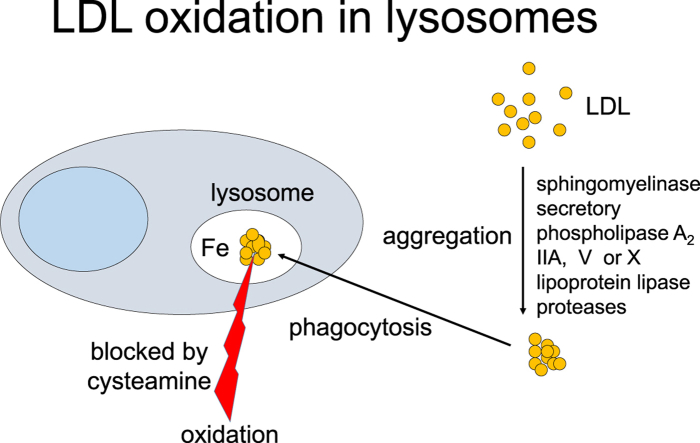

## 1 Introduction

Oxidised LDL was at one time widely believed to be important in the pathogenesis of atherosclerosis [1,2], the underlying cause of the large majority of myocardial infarctions and thrombotic strokes. This was because oxidised LDL has numerous potential pro-atherosclerotic effects. For example, even small amounts of oxidised lipids from mildly-oxidised (also called minimally-modified) LDL can activate genes for cellular adhesion molecules and chemotactic proteins [3], thus generating an inflammatory response. More highly oxidised LDL can decrease the mobility of macrophages [4], induce apoptosis [5] or necroptosis [6], increase or decrease autophagy [7] and activate the NLRP3 inflammasome [8], amongst other effects. Oxidised LDL increases the production of metalloproteinases [9], which destabilise the fibrous caps over advanced lesions causing thrombosis.

The oxidised LDL hypothesis proposes that cells in the arterial wall oxidise LDL in the extracellular space and then take it up rapidly, leading to the formation of foam cells which are characteristic of atherosclerosis [1]. Oxidised LDL has been detected in animal and human atherosclerotic lesions by immunohistochemistry [[10], [11], [12]]. Despite intensive research focusing on this hypothesis for decades, the underlying mechanisms by which LDL is oxidised in the arterial intima remain uncertain. Two key problems with this conventional view are that oxidation is inhibited by low concentrations of interstitial fluid or serum (components of which enter the wall) [13,14] and that large clinical trials have failed to show that antioxidants protect against cardiovascular disease [15].

To account for these problems, we hypothesised that LDL nonoxidatively modified and aggregated by enzymes, such as sphingomyelinase [16], proteases [17] or secretory phospholipase A_2_ enzymes [18], in the extracellular space of atherosclerotic lesions [19] is rapidly endocytosed by macrophages and delivered to lysosomes, where it might be oxidised [20]. In support of this view, we showed that 7 days after taking up mechanically-aggregated (vortexed) LDL, mouse J774 macrophage-like cells and human monocyte-derived macrophages (HMDM) generated ceroid in their lysosomes. Ceroid (lipofuscin) is a final product of lipid oxidation that consists of insoluble polymerised lipid and protein complexes and it is found within foam cells in atherosclerotic lesions [21]. Furthermore, 7-ketocholesterol, one of the main products of LDL oxidation, was detected by HPLC after J774 cells were treated with acetylated LDL, which is also rapidly endocytosed. Chloroquine, a weak base that concentrates in lysosomes and increases their pH, inhibited the oxidation of LDL, consistent with lysosomes being the critical intracellular organelle [20].

We further proposed that the oxidation in lysosomes is mediated by iron [20]. It is known that foam cells in human atherosclerotic lesions contain catalytically active iron in their lysosomes [22,23]. We have demonstrated that iron [20,24,25] or ferritin [26] can oxidise LDL effectively at the lysosomal pH of 4.5 (but not at pH 7.4), as measured by spectrophotometry and the iron chelator desferrioxamine, which is pinocytosed by cells and delivered to lysosomes, inhibited LDL oxidation in the experiments described above [20]. The oxidation of LDL by iron at acidic pH is inhibited by cysteamine (2-aminoethanethiol) [27], an antioxidant that accumulates in lysosomes [28]. The lysosomal oxidation of LDL in the lysosomes of macrophages increases their pH and causes the secretion of inflammatory cytokines, both of which are decreased by cysteamine [27].

Collectively, these findings support our explanation of how LDL is oxidised in atherosclerotic lesions despite the presence of numerous antioxidants in interstitial fluid. They also suggest why large clinical trials have shown no protection against cardiovascular disease by antioxidants [15], as most antioxidants do not have ready access to lysosomes. The antioxidant α-tocopherol (vitamin E) is contained within LDL and would therefore be delivered to lysosomes with LDL, but we have shown that α-tocopherol in LDL does not inhibit LDL oxidation at acidic pH as effectively as it does at pH 7.4 [24]. The antioxidant probucol does not inhibit the initial oxidation of LDL by iron at lysosomal pH [25]. Importantly, our findings suggest new ways of inhibiting the disease process.

Our original experiments used LDL that had been aggregated by vortexing or modified by acetylation, rather than by components of the arterial wall, however, and they were only conducted *in vitro* [20]. Sphingomyelin is one of the phospholipids present in LDL. Sphingomyelinase is a sphingomyelin-specific form of phospholipase C, which hydrolyses the phosphodiester bond of sphingomyelin to generate ceramide and phosphocholine [16]. Ceramide is hydrophobic and causes the aggregation of LDL particles [29], but interactions between newly exposed segments of apolipoprotein B-100 may also be involved [30]. Sphingomyelinase can be secreted by macrophages and endothelial cells [31] and is active in the arterial wall [32], with increased sphingomyelinase activity in atherosclerotic lesions compared to normal arteries [33]. Sphingomyelinase greatly enhances subendothelial LDL retention in atherosclerotic lesions and increases lesion development [34]. The levels of ceramide are significantly elevated in atherosclerotic lesions and LDL isolated from these lesions [32,33,35].

Here we show that LDL aggregated by sphingomyelinase (SMase-LDL), a more pathophysiological form of modified LDL than vortexed or acetylated LDL, is rapidly taken up and oxidised in the lysosomes of human macrophages. We also report the effects of cysteamine and other antioxidants on this oxidation. We examined the effects of cysteamine not only on cell-free and cell-mediated oxidation *in vitro*, but also in an *in vivo* animal model of atherosclerosis in which LDL is raised.

## 2 Materials and methods

### 2.1 Chemicals and reagents

Chemicals and reagents were purchased from Sigma-Aldrich, Dorset, UK, or Fisher Scientific Ltd, Loughborough, UK, unless otherwise stated. Solutions were prepared using ultrapure water generated from a Barnstead Nanopure system. Cell culture media and serum were obtained from Life Technologies Ltd (Invitrogen, Paisley, UK). Amifostine and 2-[(3-aminopropyl) amino] ethanethiol dihydrochloride (WR-1065) were kindly supplied by the National Institute of Health, National Cancer Institute, Bethesda, Maryland, USA. Organic solvents were HPLC or molecular biology grades.

### 2.2 Aggregation of LDL with sphingomyelinase

Native LDL (1.019–1.063 g/ml) was isolated from plasma of healthy volunteers by sequential density ultracentrifugation [36]. Native LDL was diluted to 2 mg protein/ml with a buffer containing NaCl (150 mM), MgCl_2_ (10 mM) and HEPES (5 mM), pH 7.4 and incubated with sphingomyelinase from Bacillus cereus (Sigma, catalogue number S9396–25UN) at 10 mU/ml, as described by Walters and Wrenn [29,37], until the attenuance (absorbance plus light scattering) at 680 nm in a spectrophotometer, as a measure of LDL aggregation [38], increased from 0.0017 ± 0.0005 to 0.027 ± 0.005 (mean ± SEM of 4 independent experiments). Sphingomyelinase aggregated-LDL (SMase-LDL) was dialysed against phosphate buffer (140 mM NaCl, 8.1 mM Na_2_HPO_4_, 1.9 mM NaH_2_PO_4_ and 100 μM EDTA), pH 7.4 (which had been pre-treated with washed Chelex-100 to remove contaminating transition metals) and sterilised with a 0.45 μm Minisart filter before adding to the cell culture. Aggregation was confirmed by dynamic light scattering in UV grade cuvettes with a Zetasizer Nano Series particle sizer (Malvern Instruments, Worcestershire, UK).

### 2.3 Measurement of hydroperoxides

Hydroperoxides in LDL were measured by a tri-iodide assay [39].

### 2.4 Spectrophotometric measurements of LDL oxidation by iron

Native LDL (50 μg protein/ml) was incubated with FeSO_4_ (5 μM) at pH 4.5 (150 mM NaCl/10 mM sodium acetate buffer) at 37 °C in an automated spectrophotometer and the attenuance against reference cuvettes was monitored at 234 nm [24,40].

### 2.5 Cell culture

Human macrophages or THP-1 cells were cultured under humidified 95% air/5% CO_2_ at 37 °C in Gibco RPMI 1640 containing l-glutamine (0.3 g/l), penicillin (50 IU/ml), streptomycin (50 μg/ml), amphotericin B (0.95 μg/ml) and human or fetal bovine serum (10%, v/v), respectively, unless otherwise stated. THP-1 cells were purchased from the European Collection of Cell Cultures (Salisbury, UK). The THP-1 cells were differentiated into macrophages on 18 × 18 mm class coverslips in 6-well tissue culture plates using phorbol 12-myristate 13-acetate (25 ng/ml) over 3 days prior to experiments. Human monocyte-derived macrophages (HMDM) were prepared from blood donated by healthy adults using Lymphoprep™ density gradient solution (Axis-Shield, Oslo, Norway) as previously described [41]. Briefly, after separation from blood cells, monocytes were incubated in RPMI medium without serum in nonadherent 6-well tissue culture plates for 40 h, then transferred to RPMI with 10% (v/v) human serum in ordinary 6-well tissue culture plates for 10–14 days, with granulocyte macrophage colony-stimulating factor (25 ng/ml) present for just the first 3–4 days. The medium was changed every 3–4 days. Before use, the HMDM were removed with trypsin (0.25%) and cell scrapers and transferred on to coverslips at 5 × 10^6^ cells/ml. The suspension of THP-1 cells contained 1.5 × 10^5^ cells/ml. Lipoprotein-deficient serum (LPDS) was prepared by ultracentrifugation at 115 000 g and 4 °C for 48 h at the density of 1.25 g/ml.

### 2.6 Detection and quantification of ceroid in macrophages

HMDM cultured on glass coverslips were incubated in medium containing either SMase-LDL or native LDL at 200 μg protein/ml, or without LDL, for 24 h. The medium was washed off 3 times with warm PBS and the incubation was continued for 7 days with RPMI 1640 medium containing 10% (v/v) lipoprotein-deficient serum (LPDS). The medium was changed every 2 days. To study effects of antioxidants, butylated hydroxytoluene (BHT; freshly dissolved in ethanol), amifostine or WR-1065 (both freshly dissolved in nanopure water), were added to the cells every day after the LDL was washed off. To demonstrate ceroid, cells on coverslips were fixed with 4% (w/v) paraformaldehyde in PBS, treated with ethanol and xylene for 5 min each to remove ‘soluble lipids’ and stained with Oil Red O. Staining was detected using a transmitted-light microscope equipped with a digital camera (Axioskop 2, Carl Zeiss Ltd). It was quantified with ImageJ 1.46 (National Institute of Mental Health, Bethesda, Maryland, USA) by calculating the average intensity of red pixels in five randomly positioned digital images containing a total of at least 100 cells on each slide.

### 2.7 Colocalisation of intracellular lipids or ceroid with lysosomes

HMDM were incubated with SMase-LDL for either 24 h to demonstrate the lysosomal localisation of lipids or incubated for an additional 7 days in the absence of lipoproteins, as described above, to show the colocalisation of ceroid and lysosomes. After incubation with SMase-LDL, cells were washed and fixed with 4% (w/v) paraformaldehyde in PBS (pH 7.4). Lysosomes were incubated with rabbit polyclonal IgG anti-lysosomal associated membrane protein 2 antibody (anti-LAMP2 antibody, Santa Cruz Biotechnology Inc, Dallas, Texas, USA; catalogue number H207) at 200 μg/ml overnight at 4 °C, followed by incubation with Alexa Fluor 488-conjugated donkey anti-rabbit IgG (Invitrogen; catalogue number A-21206) as the secondary antibody for 1 h at room temperature. Intracellular lipids were stained with LipidTOX™ Red (Invitrogen) for 30 min at room temperature. Fluorescence was detected with a confocal microscope (Leica DMIRE2) using sequential acquisition. Excitation wavelengths of 488 nm and 594 nm were used for lysosomes and lipids, including ceroid, respectively. Lysosomal ceroid was examined after treatment with ethanol and xylene, to remove the ‘soluble lipids’.

### 2.8 HPLC analysis

Cholesterol and cholesteryl esters were quantified by reverse-phase HPLC. Lipids were extracted from macrophages using methanol and hexane [42]. The upper hexane layer was collected and evaporated at ambient temperature in a SpeedVac Concentrator System (ThermoFisher). The residue was redissolved in the mobile phase (acetonitrile/propan-2-ol (30/70, v/v)) and injected into a C18 column in an HPLC (PerkinElmer 200 series) [42]. Cholesterol and cholesteryl esters were detected at 210 nm. The identities of the peaks were confirmed by mass spectrometry (results not shown) and quantified using standards of pure chemicals from Sigma.

Cysteamine in mouse plasma samples cells was measured using 7-fluorobenzo-2-oxa-1,3-diazole-4-sulfonate by reverse-phase HPLC with a C18 column and a fluorescent detector [43]. Tri-*n-*butylphosphine was used to release protein-bound thiols and reduce oxidised thiols.

### 2.9 Animal experiments

All procedures complied with the Animals (Scientific Procedures) Act 1986 and were approved by the Ethical Review Process Committee of Imperial College London. Ten week-old LDL receptor-knockout (*LDLr*^*−/−*^) female mice (Charles River, Margate, UK) were fed normal laboratory chow for a week and then a cholate-free high fat diet (Diet W, SDS, Horley, Surrey, UK) containing cocoa butter (15%, w/w) and cholesterol (0.25%, w/w) for 12 weeks. Animals (n = 19–22 per group) received cysteamine hydrochloride at 2.2 and 8.8 mM in purified drinking water of electrical resistivity 15 MΩ-cm (equivalent to 42 mg of the free base/kg body weight/day and 170 mg/kg body weight/day, assuming 20 g mice drink 5 ml of water per day [44]) or purified drinking water as a control. Water was changed daily. At the end of the trial, mice were weighed and killed by pentobarbital overdose (20 mg *ip*). Blood was taken by cardiac puncture with EDTA as the anticoagulant, the chest cavity was cut open to expose the heart and 10 ml of PBS was perfused through the circulatory system via the left ventricle, draining through a cut in the right atrium. The animals were then stored in 2% (w/v) paraformaldehyde in NaCl (150 mM) at 4 °C for ≥48 h before dissection. Blood samples were centrifuged and plasma aliquots were stored at −80 °C before measurement of cysteamine by HPLC [43] and lipid profile with an ILab600 chemical analyser using kits supplied by Instrumentation Laboratory [45].

### 2.10 Lesion measurement

The heart, aortic root, aortic arch and descending aorta were dissected as previously described [46,47]. The specimens were coded and analysed blindly regarding the treatment groups. The hearts were embedded in gelatin and frozen in OCT embedding medium. The aortic root was serially sectioned at 10 μm from where the aortic sinus appeared until the point where valve bases were shrunken, but still visible. Four sections per slide were saved, resulting in a total of 20 slides saved. Every other slide was stained with Oil Red O, Harris haematoxylin and Light Green, as described by Baglione and Smith [48]. The aortic segment from the arch to the iliac bifurcation was cut open under a dissecting microscope, pinned to a silicone elastomer (Sylgard 184, Dow Corning) in a Petri dish and stained with Oil Red O. Images were acquired with a digital camera fitted to a dissecting microscope. Lesions were quantified by drawing around the digital images using ImageJ and measuring their areas, using a scale with a known distance. Their extent was expressed as μm^2^ cross sectional area in the aortic root and per cent *en face* area covered by lesions in the aortic arch and in the rest of the thoracic plus abdominal aorta. The lesion area in the aortic root of each mouse was calculated as the mean for the ten slides that were quantified.

### 2.11 Statistical analysis

Data are presented as mean ± SEM of at least 3 independent experiments or of 19–22 mice within each group. Comparisons between control and treated samples were analysed by one-way-ANOVA with Dunnett's or Tukey's *post hoc* tests or, where appropriate, by t-tests. Differences were considered significant at *p* < 0.05.

## 3 Results

### 3.1 Aggregation of LDL by sphingomyelinase

Incubation of native LDL with sphingomyelinase for 4 h at 37 °C increased the LDL particle diameter from 26 ± 2 nm to 170 ± 28 nm (Supplementary Fig. 1A). The increase in LDL particle size caused an increase in light scattering at 680 nm, as monitored in a spectrophotometer (Supplementary Fig. 1B). Although the particle size might continue to increase with time, the optimal average LDL particle diameter was considered to be about 200 nm, as larger particles may be lost when sterilising with a 0.45 μm filter. No hydroperoxides were detected by a tri-iodide assay in SMase-LDL (or native LDL) in two independent experiments. In another experiment, the hydroperoxides in SMase-LDL were 7.8 nmol/mg protein (and were undetectable in native LDL). This was only about 1% of the maximum levels found in oxidised LDL [49]. LDL aggregation by sphingomyelinase was not affected by cysteamine (see below) (Supplementary Fig. 2).

### 3.2 Accumulation of LDL in lysosomes in macrophages

**Fig. 1:**
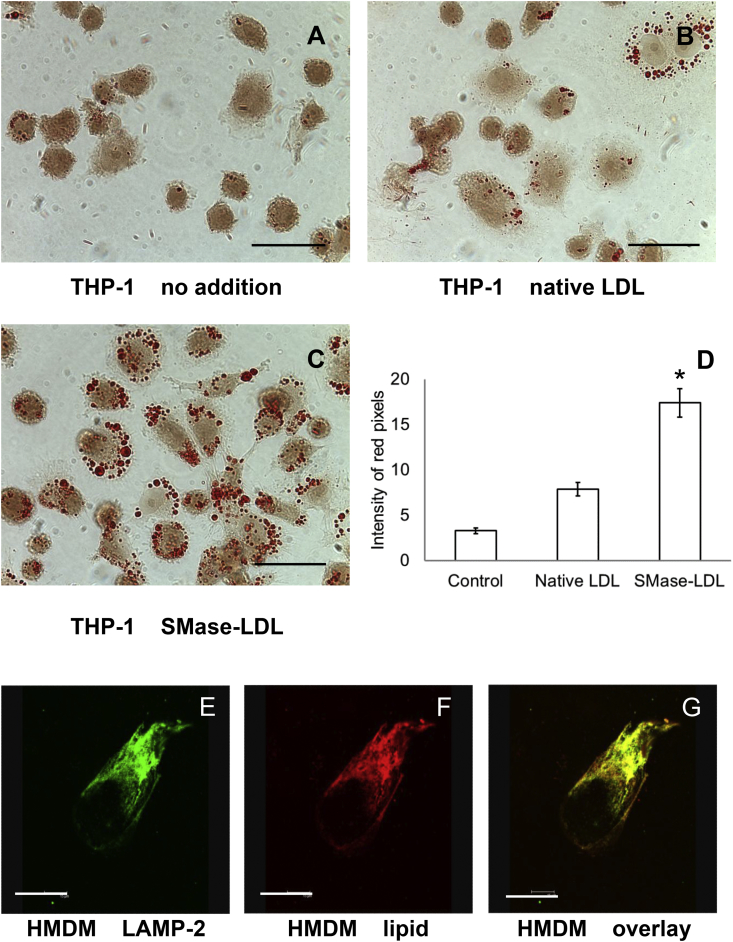
Lipids from SMase-LDL rapidly accumulate in human macrophages and colocalise with lysosomes. THP-1 macrophage-like cells and HMDM cells were incubated with SMase-LDL (200 μg protein/ml) for 4 or 24 h, respectively. The uptake of aggregated LDL by cells was detected by Oil Red O staining and transmitted-light microscopy. THP-1 macrophages were incubated (A) alone, (B) with native LDL or (C) SMase-LDL. Scale bar: 30 μm. (D) Intracellular lipids in THP-1 macrophage-like cells, measured as intensity of red pixels, were quantified using ImageJ. Mean ± SEM of 3 independent experiments. *p* < 0.001 ANOVA; followed by Tukey's *post-hoc* test, *p < 0.01 compared to others. Lysosomes in HMDM were labelled with anti-LAMP2 antibody (E) whilst intracellular lipids were stained with LipidTOX™ Red (F). Their colocalisation appeared yellow in an overlaid confocal image (G). Scale bar: 30 μm.

Incubation with SMase-LDL for 4 h caused substantial lipid-accumulation in THP-1 macrophages-like cells, with SMase-LDL causing significantly more lipid accumulation than native LDL (Fig. 1A–D). Intracellular lipids colocalised with lysosomes labelled with anti-LAMP2 antibody in HMDM (Fig. 1E–G).

HPLC [42,50] showed that THP-1 cells incubated with SMase-LDL for 24 h had greatly increased levels of cholesteryl linoleate and palmitate (Supplementary Table 1). These lipids were very low or absent in control cells. Nonesterified cholesterol doubled in cells incubated with SMase-LDL.

### 3.3 Ceroid formed in lysosomes of macrophages

**Fig. 2:**
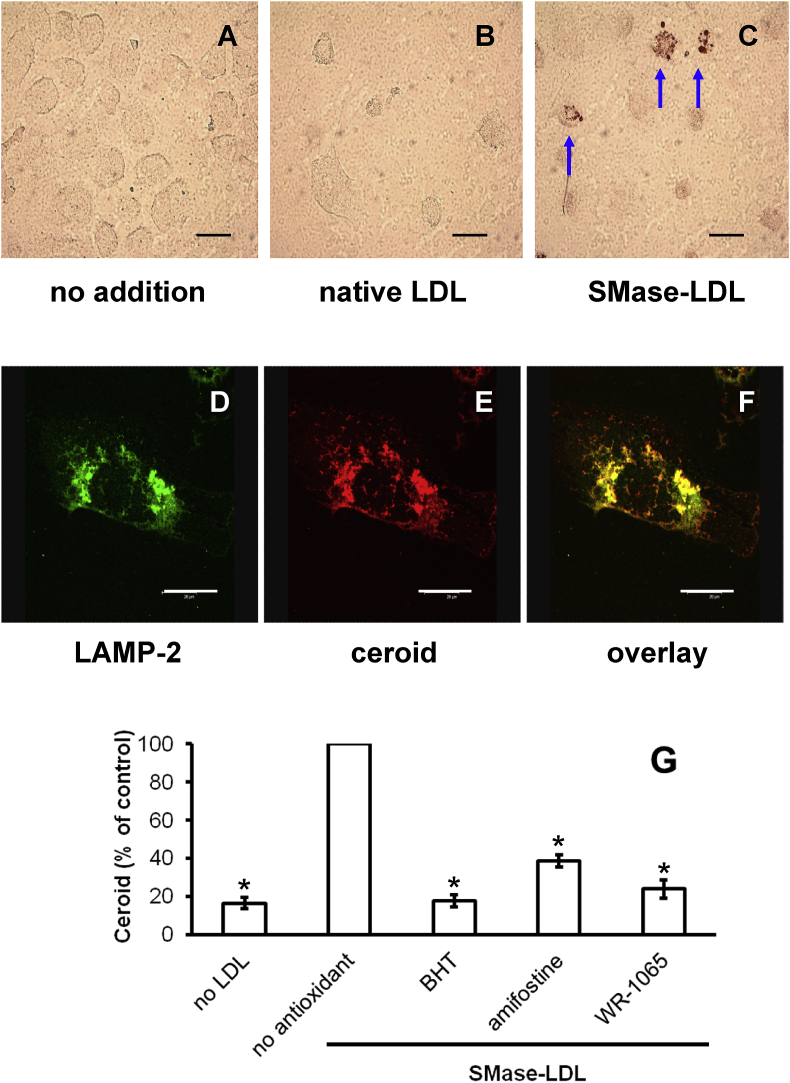
Human macrophages incubated with SMase-LDL generate lysosomal ceroid. HMDM cells were incubated without LDL (A), with native LDL (B) or SMase-LDL (C) (200 μg protein/ml) in 45% RPMI medium and 45% buffer (10 mM MgCl_2_, 5 mM HEPES and 150 mM NaCl, pH 7.4) with 10% (v/v) human serum for 24 h. The cells were then cultured for a further 7 days in RPMI with 10% (v/v) LPDS. The cells were stained for ceroid (A, B and C). Scale bar: 30 μm. Colocalisation of ceroid and lysosomes was observed by confocal microscopy for cells incubated with SMase-LDL. (D) Lysosomes were labelled with an anti-LAMP2 antibody, (E) ceroid was stained with LipidTOX™ Red and (F) an overlaid image is shown. Scale bar: 30 μm. HMDM were incubated with SMase-LDL (200 μg protein/ml) for 24 h. They were then incubated for a further 7 days in the absence of lipoproteins in RPMI with 10% (v/v) LPDS, but in the presence of BHT (10 μM), amifostine (100 μM) or WR-1065 (100 μM). Cells were also incubated without LDL or antioxidants. The ceroid levels in the cells were quantified using ImageJ to show inhibition by BHT, amifostine and WR-1065. Mean ± SEM of 3 independent experiments. *p < 0.001, ANOVA and *post-hoc* Dunnett's test compared to SMase-LDL with no antioxidant.

HMDM or THP-1 macrophage-like cells were cultured in RPMI containing human serum alone or also containing native or SMase-LDL for 24 h and were washed and incubated for 7 days in the complete absence of lipoproteins. The lipid content of THP-1 cells was increased in a concentration-dependent manner by SMase-LDL, as shown by Oil Red 0 staining (Supplementary Fig. 3). The cells were incubated in the absence of lipoproteins during the 7-day ‘chase’ period to exclude the possibility that lipoproteins are oxidised in the medium during this time and taken up by the cells. Any oxidation of lipoproteins would therefore have to be intracellular. Ceroid was clearly visible in HMDM in the form of Oil Red O stained, irregularly shaped granules in cells treated with SMase-LDL after other lipids had been removed by organic solvents [21] (Fig. 2C). No significant ceroid was present in cells that had been incubated without LDL (Fig. 2A) or with native LDL (Fig. 2B). LipidTOX™ Red stained ceroid colocalised with anti-LAMP2-labelled lysosomes in cells incubated with SMase-LDL (Fig. 2D–F). Ceroid was also present in THP-1 cells incubated under similar conditions (Supplementary Fig. 4).

We next investigated the effects of selected lipophilic and hydrophilic antioxidants on lysosomal LDL oxidation. Lysosomal lipid accumulation was induced in HMDM using SMase-LDL, which was then washed off and the incubation continued for 7 days in the complete absence of lipoproteins but in the presence of the lipophilic antioxidant BHT and the hydrophilic prodrug amifostine, which is converted by alkaline phosphatase *in vivo* into the antioxidant WR-1065. The structures of these antioxidants are shown in Supplementary Fig. 5. All these compounds inhibited ceroid formation in HMDM considerably (Fig. 2G).

### 3.4 LDL oxidation by iron at lysosomal pH is inhibited by cysteamine

**Fig. 3:**
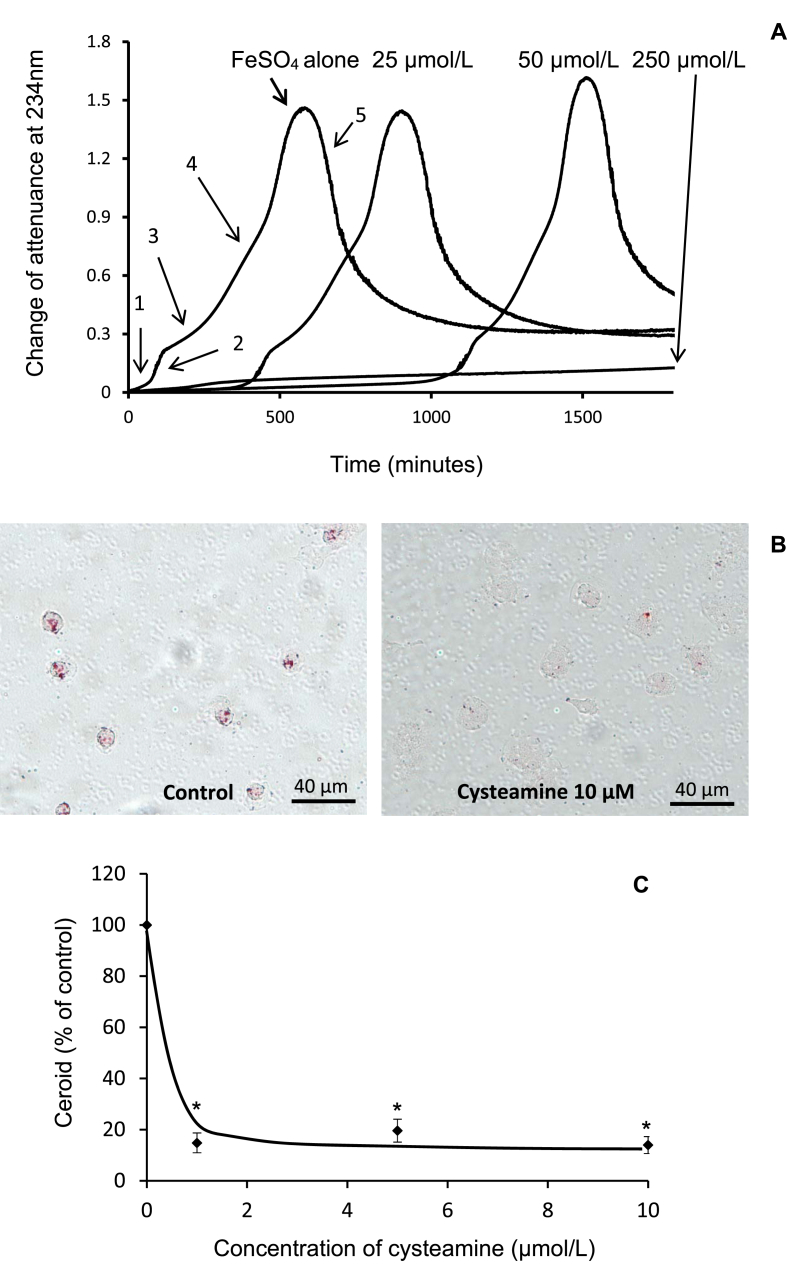
Cysteamine inhibits LDL oxidation by iron at pH 4.5 and ceroid formation in macrophages. (A) LDL (50 μg of protein/ml) in NaCl/sodium acetate buffer (pH 4.5) was incubated with 5 μM FeSO_4_ at 37 °C in quartz cuvettes. Cysteamine (final concentrations 25, 50 and 250 μM) was added to the cuvettes at the start of the incubation. Oxidation and UV scattering were monitored by measuring the change in attenuance (absorbance plus UV scattering) at 234 nm against appropriate reference cuvettes. The stages of oxidation are marked with arrows: 1, lag phase; 2, rapid oxidation phase; 3, slow oxidation phase; 4, aggregation phase and 5, sedimentation phase. These data are representative of three independent experiments. Cysteamine at 25 μM increased the time required to increase the attenuance to 0.1 by 5.5 ± 1.5 fold (*p* < 0.05) and at 50 μM by 14.4 ± 1.2 fold (*p* < 0.001), mean ± SEM of 3 independent experiments; ANOVA and Dunnett's *post-hoc* test. (B) Human monocyte-derived macrophages were cultured on coverslips and incubated with SMase-LDL at 200 μg protein/ml for 24 h. They were washed and cultured for 7 days with RPMI 1640 medium containing 10% (v/v) human lipoprotein-deficient serum to which cysteamine was added every 24 h. The cells were then washed, fixed, treated with ethanol and xylene to remove non-ceroid lipids and stained for ceroid with Oil Red O. Ceroid was quantified using ImageJ. (C) Mean ± SEM of 4 independent experiments. *p <0.001, ANOVA and Dunnett's *post hoc* test.

We explored in more detail the effects of the lysosomotropic drug cysteamine (structure given in Supplementary Fig. 5). Cysteamine inhibited the oxidation of LDL greatly and in a concentration-dependent manner when LDL was incubated with FeSO_4_ in a spectrophotometer at the lysosomal pH of 4.5 (Fig. 3A). The lag phase increased from about 1 h to over 30 h when 250 μM cysteamine was added. Cysteamine added to cultured HMDM at a concentration of 1 μM or over inhibited ceroid production by ≥ 80% (Fig. 3B and C).

### 3.5 Atherosclerosis in mice is inhibited by cysteamine

We next investigated the effect of cysteamine on atherosclerosis in LDL receptor-deficient mice fed an atherogenic diet. Two doses were used: one equivalent to that received by patients with cystinosis (42 mg free cysteamine base per kg body weight per day [51]), assuming the mice drank 5 ml of water per day [44], and a higher one in case the human dose was ineffective.

As cysteamine might be oxidised in solution, we assessed its stability in the drinking water supplied to the mice, which was purified water with a high resistivity (15 MΩ-cm), using a HPLC assay. There was a 54% and 53% loss of free cysteamine for the lower (2.2 mM) and higher doses (8.8 mM), respectively, after 24 h. We also assessed the ability of cysteamine solutions to retain their ability to inhibit the oxidation of LDL by iron. Cysteamine (10 mM) was left in the drinking water used for the mice (15 MΩ-cm) or ultrapure water (18.2 MΩ-cm) at room temperature for 48 h. When diluted 200 fold, it was still able to inhibit the oxidation of LDL by 5 μM FeSO_4_ at pH 4.5, but it was, respectively, 69 ± 3% and 34 ± 2% (mean ± SEM of three experiments) less effective than freshly made solutions at prolonging the lag phase. There would therefore have been some loss of cysteamine activity in the drinking water the mice received, which was changed every day, but most of the cysteamine should have remained intact.

No differences were observed among the control and cysteamine groups for body weight and plasma lipid and lipoprotein concentrations (Table 1).

**Table 1:** Effect of cysteamine on atherosclerosis in LDL receptor knockout mice.

	Control (n = 19)	Cysteamine (2.2 mM in drinking water) (n = 21)	Cysteamine (8.8 mM in drinking water) (n = 22)
Weight (g)	22.4 ± 0.6	22.3 ± 0.4	22.8 ± 0.7
Cholesterol (mM)	39.9 ± 2.5	40.3 ± 1.5	38.8 ± 1.9
Triacylglycerol (mM)	4.64 ± 0.31	4.67 ± 0.29	4.99 ± 0.29
HDL-cholesterol (mM)	3.30 ± 0.24	3.34 ± 0.12	3.23 ± 0.16
LDL-cholesterol (mM)	29.6 ± 1.7	29.4 ± 0.9	26.8 ± 1.1
Cysteamine (μM)	0.0933 ± 0.034	0.251 ± 0.019	0.764 ± 0.071*
Aortic root (mm^2^)^$^	0.487 ± 0.025	0.397 ± 0.029	0.397 ± 0.026
Aortic arch (%)^#^	35.0 ± 1.8	26.0 ± 1.2	27.0 ± 1.1
Thoracic + Abdominal (%)	2.29 ± 0.64	1.53 ± 0.32	1.36 ± 0.27

Body weight, plasma lipids, plasma cysteamine concentrations and atherosclerotic lesions in three aortic regions (mean ± SEM) were measured in mice fed a Western diet with or without cysteamine for 12 weeks. ANOVA showed no significant difference in body weight and lipids between groups. Cysteamine concentrations in plasma were significantly higher in mice receiving the drug compared to the control group (*p* < 0.001, ANOVA), with the higher dose group having higher plasma concentrations than the control or lower dose groups (*p < 0.0001, Tukey's *post hoc* test). The inhibition of the disease was not significantly affected by dose at any of the three aortic locations examined, as assessed by either 2-way ANOVA (*p* = 0.65) or *t*-test (*p* = 0.93 for aortic root, *p* = 0.57 for aortic arch and *p* = 0.69 for remaining thoracic plus abdominal aorta) and data for the two doses were therefore combined. The effect of cysteamine was significant in the aortic root (^$^*p* = 0.010; *t*-test) and arch (^#^*p* = 0.00001; *t*-test). It appeared to cause an even greater reduction in the descending aorta but the effect only approached statistical significance (*p* = 0.11), probably because the level of disease was low in these regions and the measurements were consequently highly variable.

**Fig. 4:**
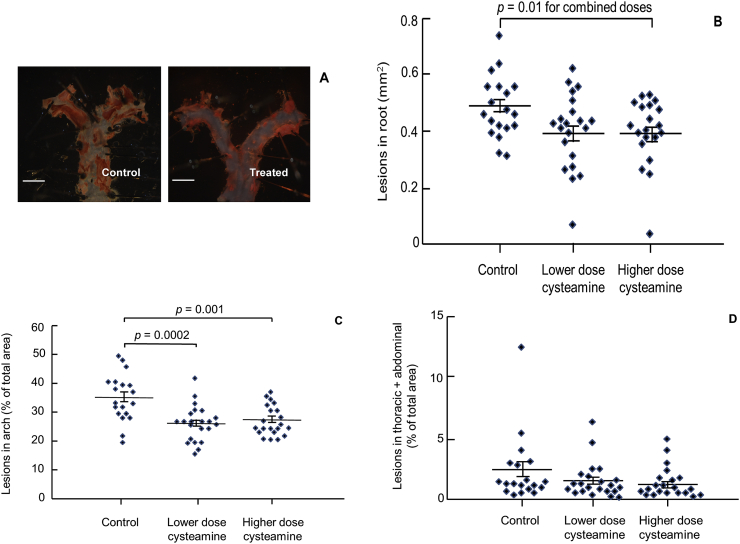
Cysteamine reduced atherosclerosis in LDL receptor-deficient mice. (A) Representative digital images to show atherosclerotic lesions in the aortic arch of control mice and mice treated with the lower dose of cysteamine (2.2 mM in drinking water). Scale bar: 2 mm. Data points to show lesion areas in individual mice in each group in aortic root (B), aortic arch (C) and the remaining thoracic plus abdominal aorta (D) of mice given the low (2.2 mM) and high doses (8.8 mM) of cysteamine. There were 19–22 mice in each group and the horizontal line shows the group mean ± SEM. The combined effect of both doses of cysteamine was significant in the aortic root (*p* = 0.010; *t*-test) and arch (*p* = 0.00001; *t*-test) and was significant for the lower dose alone (*p* < 0.001 ANOVA; *p* = 0.0002 control *vs.* lower dose, Tukey's *post hoc* test) and the upper dose alone (*p* = 0.001 control *vs.* higher dose, Tukey's) for the aortic arch.

The inhibition of disease by the lower and higher doses was, respectively, 18.4% and 18.5% in the root, 25.5% and 22.7% in the arch and 33.3% and 40.7% in the remaining thoracic plus abdominal aorta (Table 1). Lesion areas for the individual mice and representative images of aortic arches from the control and cysteamine groups are shown in Fig. 4. The reductions were not significantly affected by dose at any of the three aortic locations, as assessed by 2-way ANOVA (*p* = 0.65) or *t*-test (*p* = 0.93 for aortic root, *p* = 0.57 for aortic arch and *p* = 0.69 for the remaining thoracic plus abdominal aorta) (Table 1). The data for the two doses were therefore combined for further statistical analysis. The effect of cysteamine was significant in both the aortic root (*p* = 0.010; *t*-test) and arch (*p* = 0.00001; *t*-test). Cysteamine appeared to cause a greater reduction in the remaining thoracic and abdominal aorta, but the effect only approached statistical significance (*p* = 0.11; *t*-test), probably because the level of disease was low in this region (coverage averaging only around 2%) and the measurements were consequently highly variable (the coefficient of variation was 79%, compared to 20% in the root and 17% in the arch).

There were low, but detectable, concentrations of cysteamine in the plasma of control mice (Table 1). This is expected because cysteamine is generated in animals as part of the coenzyme A catabolic pathway [52]. The cysteamine concentrations were significantly higher in mice receiving the drug, with higher concentrations in the higher dose group. Our lower and higher dose treatments appeared to result in plasma cysteamine levels much below those reported for humans. The plasma cysteamine concentrations are around 40 μM in patients on oral cysteamine [53]. This discrepancy may be an artefact arising from the nocturnal feeding habits of mice. Cysteamine has a short half-life; about 2 h in humans [54] and 0.5 h in mice [55]. The blood of the mice was taken in the mid-morning when plasma concentrations would have fallen considerably. Even these concentrations are expected to inhibit the lysosomal oxidation of LDL in macrophages considerably (Fig, 3*C*).

## 4 Discussion

We previously reported that LDL acetylated to increase its net negative charge or aggregated by vortexing is taken up rapidly by macrophages and oxidised in lysosomes [20]. Here we describe experiments carried out with LDL aggregated (but not oxidised) by sphingomyelinase, a more pathophysiological approach to study the lysosomal oxidation of LDL, as sphingomyelinase is present in atherosclerotic lesions and may be one of the key enzymes causing the aggregation of LDL in the extracellular space of atherosclerotic lesions [34]. The susceptibility of LDL to sphingomyelinase-induced aggregation predicts future coronary heart disease deaths [56]. We found that incubation of LDL with sphingomyelinase increased the LDL particle size from 26 nm to 170 nm (Supplemental Fig. 1). Lipid accumulation in macrophages was increased greatly by SMase-LDL as shown by Oil Red O staining (Fig. 1A–D) and by HPLC (Supplementary Table 1). The levels of nonesterified cholesterol, cholesteryl linoleate and cholesteryl palmitate, were increased in macrophages incubated with SMase-LDL (Supplementary Table 1). The much higher levels of cholesteryl linoleate compared to cholesteryl oleate is consistent with the lipid being mainly lysosomal, as the main cholesteryl ester in LDL is cholesteryl linoleate whereas the main cholesteryl ester in cytosolic lipid droplets is cholesteryl oleate [57]. The lipids were localised in lysosomes as shown by confocal microscopy (Fig. 1E–G). Aggregated LDL (produced by vortexing) has been shown to be degraded in extracellular compartments on the surface of macrophages (lysosomal synapses), which contain regions of acidity [58,59], but most of our aggregated LDL was transported to lysosomes.

Importantly, LDL aggregated by sphingomyelinase oxidised to form the advanced oxidation product ceroid in lysosomes of macrophages. Ceroid is a final product of lipid oxidation that consists of insoluble polymerised lipids and proteins and is found within foam cells in human atherosclerotic lesions [21]. Ceroid is formed in lysosomes by an iron-catalysed oxidative process and its production can be diminished by antioxidants or iron chelators [20,25,60]. It can be detected as Oil Red O-stained lipid after other lipids have been removed by organic solvents [21]. The colocalisation study (Fig. 2D–F) showed clearly that lysosomes contained ceroid generated from internalised LDL. Lysosomes contain catalytically active iron [22] and iron staining is common in human advanced atherosclerotic lesions and colocalises with ceroid [23]. This iron may be derived either by autophagy of organelles, together with their iron-containing proteins, or from the endocytosis of iron-containing proteins [61].

We tested if the lysosomal oxidation of SMase-LDL that had already accumulated in lysosomes could be inhibited using antioxidants that have access to lysosomes. BHT, which contains an antioxidant phenolic hydroxyl group (Supplementary Fig. 5), inhibited the oxidation effectively (Fig. 2G). The prodrug amifostine and its active product WR-1065, which contains an antioxidant thiol group (Supplementary Fig. 5), also inhibited the lysosomal oxidation of SMase-LDL effectively (Fig. 2G). Amifostine protects normal tissues from damage caused by radiation and chemotherapy [62]. WR-1065 protects macrophages against death induced by hydrogen peroxide, which is mediated by lysosomal redox active iron [62]. The prodrug amifostine would have to be hydrolysed to WR-1065 before uptake, presumably by alkaline phosphatase, which is present on human-monocyte-derived macrophages [63]. The active drug WR-1065 may diffuse into lysosomes and then become protonated and trapped in these organelles, accumulating to high concentrations, as do other lysosomotropic compounds.

Lysosomal oxidation of LDL in HMDM was also inhibited by the lysosomotropic drug cysteamine (Fig. 3B and C), which was effective at very low concentrations due to accumulation within lysosomes [28]. LDL oxidation by iron at lysosomal pH was also inhibited by cysteamine, with a greatly extended lag period (Fig. 3A).

Importantly, atherosclerosis in both the aortic root and arch of LDL receptor-deficient mice on a Western diet was highly significantly reduced by cysteamine, in the absence of any changes in plasma lipoprotein concentrations (Table 1). One possible mechanism by which cysteamine protects against atherosclerosis is by inhibiting the lysosomal oxidation of LDL. The lower dose, equivalent to that given to cystinosis patients [51], was as effective as the higher dose (Fig. 4), presumably because the accumulation of cysteamine in lysosomes meant that the lower dose was sufficient to inhibit effectively the oxidation of LDL in these organelles. Cysteamine does not inhibit the aggregation of LDL by sphingomyelinase (Supplementary Fig. 2) but should inhibit the lysosomal oxidation of LDL. It might therefore have more effects on the inflammatory and toxic effects of oxidised LDL than on lipid accumulation in the arterial wall. We have recently shown that cysteamine inhibits the secretion of pro-inflammatory cytokines from macrophages incubated with SMase-LDL by inhibiting the lysosomal oxidation of LDL [27].

These results support our hypothesis that the lysosomal oxidation of LDL is a critical underlying cause of atherosclerosis and hence antioxidant drugs that concentrate in lysosomes might well provide a novel therapy for this disease. The rare lysosomal storage disorder cystinosis is an inherited disease caused by the absence of functional cystinosin, the ubiquitous lysosomal cystine transporter [64]. The consequent accumulation of cystine in the lysosomes of all cells in the body leads to progressive dysfunction of multiple organs. Cysteamine reacts with cystine to form the mixed disulfide of cysteamine and cysteine, which can leave the lysosome via the lysine/arginine transport system [65]. The therapy, considered the only effective treatment for cystinosis, prolongs the patients’ lives from about 10 years to as much as 40 years [66]. Cysteamine may also be a potential treatment for neuronal ceroid lipofuscinoses, a group of inherited progressive neurodegenerative disorders [67]. Cysteamine is given to cystinosis patients orally several times a day from childhood indefinitely. The plasma concentrations in patients are well above those needed in culture medium to inhibit lysosomal LDL oxidation inside human macrophages, measured by the formation of the advanced oxidation product ceroid (Fig. 3). The effectiveness of cysteamine at these extracellular concentrations may reflect its accumulation in lysosomes. The most common adverse effects of cysteamine [52] are gastrointestinal problems, which can be treated by proton pump inhibitors, and unpleasant breath and sweat odour, which might possibly be avoided by using prodrugs [68].

Arterial calcification is frequently a sequela of atherosclerosis. Our hypothesis is therefore supported by the observation that there is a striking inverse relationship between arterial calcification and the number of years that cystinosis patients have been on cysteamine and a striking positive relationship between arterial calcification and the number of years that they have been off cysteamine [69]. A clinical trial using cysteamine to try to prevent cardiovascular disease would be a far better test of the oxidised LDL hypothesis of atherosclerosis than ones using α-tocopherol, which is a far less effective antioxidant for LDL at lysosomal pH than pH 7.4 [24].

## Financial support

This study was supported by the British Heart Foundation (Project Grant numbers PG/10/016 and PG/15/98/31864).

## Author contributions

YW and FA performed the *in vitro* experiments, atherosclerotic lesion measurements and data analysis. ZM and YW ran the *in vivo* experiments and dissected the mice. DSL and PDW designed the study and wrote the grant application. All authors were involved in writing the manuscript.

## Declaration of competing interest

The authors declared they do not have anything to disclose regarding conflict of interest with respect to this manuscript.
