# Supplementary material for: Cysteamine inhibits lysosomal oxidation of low density lipoprotein in human macrophages and reduces atherosclerosis in mice
Source: Atherosclerosis. 2019 Dec;291:9–18. doi: 10.1016/j.atherosclerosis.2019.09.019 (PMC6912160; doi:10.1016/j.atherosclerosis.2019.09.019)
Supplement: Multimedia component 1 [file mmc1.docx]

**Supplementary Material for online publication only**

**Time of incubation (min)**

**Particle size (nm)**

**Control LDL**

**SMase-LDL**

**A**

**B**

**Supplementary Fig. 1.** Aggregation of LDL by sphingomyelinase.

LDL (2 mg protein/ml) was incubated at 37° C with sphingomyelinase (10 mU/ml). (A) LDL particle size was measured by dynamic light scattering. (B) The increase in LDL particle size caused an increase in attenuance (absorbance plus light scattering) at 680 nm (measured after dilution to 100 µg LDL protein/ml) monitored in a spectrophotometer. Both are the mean ± SEM of 3 independent experiments.

A

B


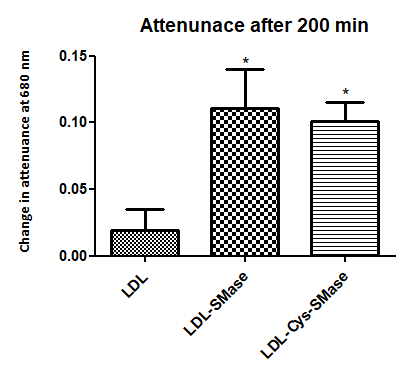


**Supplementary Fig. 2.** Cysteamine does not affect the aggregation of LDL by sphingomyelinase.

(A) Native LDL (50µg protein/ml) was incubated at 37° C either alone or with sphingomyelinase (10 mU/mL) or sphingomyelinase (10 mU/mL) and cysteamine (10 µM) in a buffer containing NaCl (150 mM), MgCl_2_ (10 mM) and HEPES (5 mM), pH 7.4. The increase in LDL aggregation was measured using light scattering at 680 nm in a spectrophotometer. (B) The change in attenuance after 200 min is shown (**p*<0.05, ANOVA followed by Tukey’s post-hoc test, n=3 independent experiments).


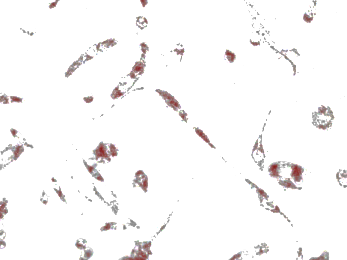

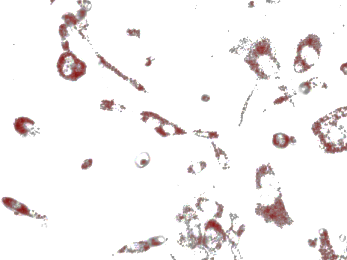


**B**

**A**


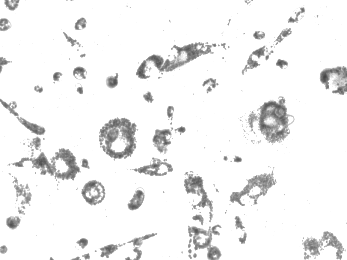

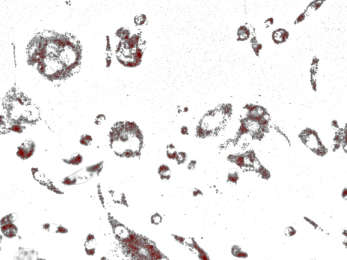


**C**

**D**


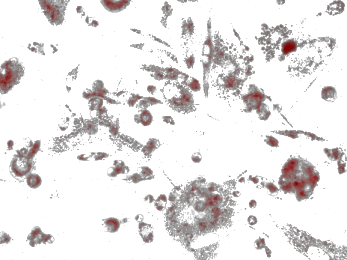


**E**

**F**

**Supplementary Fig. 3.** Sphingomyelinase-aggregated LDL causes lipid accumulation in macrophages.

THP-1 macrophage-like cells were incubated without LDL (A) or with native LDL (200 µg protein/ml) (B) or SMase-LDL (50, 100 or 200 µg protein/ml) (C-E) for 24 h. They were then incubated for a further 7 days in the absence of lipoproteins in RPMI with 10% (v/v) LPDS. The cells were then stained for intracellular lipids with Oil Red O. The lipid levels in the cells were quantified using ImageJ as mean integrated density of at least 100 cells (F). Mean ± SEM of 3 independent experiments. **p*<0.05, ****p*< 0.001 compared to No LDL by ANOVA and post-hoc Dunnett’s test; ^##^*p*<0.01, ^###^*p*<0.001 for the indicated comparison by ANOVA and post-hoc Tukey’s test.

**C**


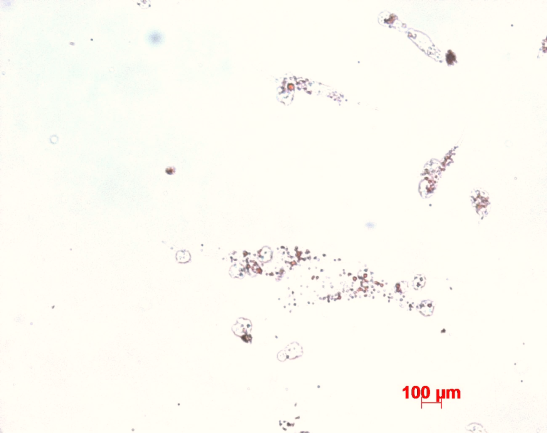

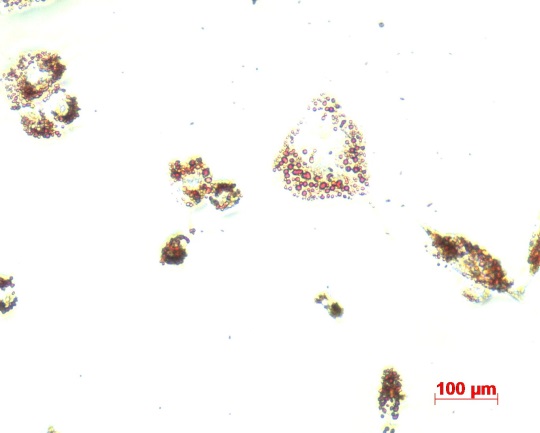


**A**

**B**

**C**

**Supplementary Fig. 4.** Sphingomyelinase-aggregated LDL causes ceroid formation in macrophages.

THP-1 macrophages grown on cover slips were incubated with SMase-LDL (200 µg protein/ml) for 24 h. They were then incubated for a further 7 days in the absence of lipoproteins in RPMI with 10% (v/v) LPDS. The cells were then stained for either ceroid or total intracellular lipids. The levels of lipids and ceroid in the cells were quantified using ImageJ as percent area of at least 100 cells. Mean of 2 independent experiments.

[
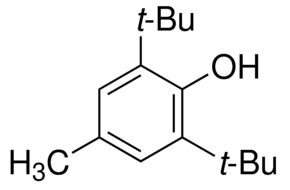
](http://www.sigmaaldrich.com/catalog/product/aldrich/w218405?lang=en&region=GB) [
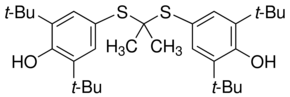
](http://www.sigmaaldrich.com/catalog/product/fluka/p9672?lang=en&region=GB)

BHT probucol

[
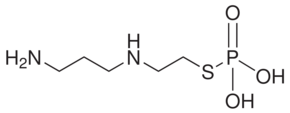
](http://www.sigmaaldrich.com/catalog/product/sigma/a5922?lang=en&region=GB) [
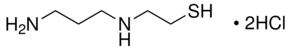
](http://www.sigmaaldrich.com/catalog/product/sigma/w2020?lang=en&region=GB)

amifostine WR-1065


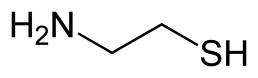


cysteamine

**Supplementary Fig. 5.** Chemical structures of BHT, amifostine, WR-1065 and cysteamine.

Amifostine is converted to WR-1065 by alkaline phosphatase *in vivo*.

**Supplementary Table 1.**

Cholesterol and cholesteryl esters in THP-1 macrophage-like cells incubated with SMase-LDL

| Treatment | Chol* | CP* | CO | CL** | CA |
| --- | --- | --- | --- | --- | --- |
| Control 0 h | 4.31±0.94 | 0.01±0.01 | 0 | 0.12±0.12 | 0.28±0.27 |
| Control 24 h | 3.89±0.95 | 0.02±0.02 | 0.01±0.01 | 0.19±0.15 | 0.43±0.41 |
| Native LDL 24 h | 4.30±0.70 | 0.02±0.02 | 0.02±0.02 | 0.18±0.17 | 0.44±0.42 |
| SMase-LDL 24 h | 8.39±0.89^#^ | 1.06±0.46^#^ | 0.50±0.25 | 4.23±0.91^#^ | 1.82±0.78 |

Cholesterol and cholesteryl esters were measured by HPLC in THP-1 macrophage-like cells incubated for 24 h without LDL (control) and with native LDL or SMase-LDL (200 µg protein/ml). The values are given as nmol/mg cell protein. Chol: nonesterified cholesterol; CP: cholesteryl palmitate; CO: cholesteryl oleate; CL: cholesteryl linoleate; CA: cholesteryl arachidonate. Mean ± SEM of 3 independent experiments. One-way ANOVA **p*<0.05, ***p*=0.001 indicated at the top of columns; Tukey’s test ^#^*p*<0.05 compared to all the values above it in the column.
